# A novel mutation within the lactase gene (*LCT*): the first report of congenital lactase deficiency diagnosed in Central Europe

**DOI:** 10.1186/s12876-015-0316-0

**Published:** 2015-07-28

**Authors:** Walid Fazeli, Sigrid Kaczmarek, Martin Kirschstein, René Santer

**Affiliations:** 1 Department of Paediatrics, University Medical Center Hamburg-Eppendorf, Martinistraße 52, D-20246 Hamburg, Germany; 2 Department of Paediatrics, General Hospital Celle, Celle, Germany

**Keywords:** Congenital lactase deficiency, CLD, *LCT* gene, Nephrocalcinosis

## Abstract

**Background:**

Congenital lactase deficiency is an extremely rare gastrointestinal disorder characterized by neonatal-onset watery diarrhoea and failure to thrive. We present the first genetically confirmed case of congenital lactase deficiency in Central Europe.

**Case presentation:**

After an uneventful pregnancy and birth, a male newborn of consanguineous parents of Turkish origin presented with watery diarrhoea. On day 17, he was admitted to hospital with weight loss, hypertonic dehydration, and metabolic acidosis. Additionally, the patient showed an elevated calcium concentration in blood and urine as well as nephrocalcinosis. Diarrhoea stopped during intravenous rehydration and when feeding a glucose-, galactose-, and lactose-free formula. Therefore, glucose-galactose-malabsorption was assumed. However, genetic testing of the *SGLT1* (*SLC5A1*) gene was negative and, indeed, feeding maltodextrine did not result in recurrence of diarrhoea. In contrast, lactose feeding immediately caused watery diarrhoea, suggesting congenital lactase deficiency. Genetic testing of the *LCT* gene revealed homozygosity for a 1-bp deletion in exon 8 (c.3448delT). Because of the nature of the mutation, causing a frame shift and a premature termination of translation, congenital lactase deficiency was confirmed and intestinal biopsies were unnecessary. The patient’s general condition improved substantially on a lactose-free diet, including hypercalcaemia, hypercalciuria, and nephrocalcinosis which, however, only disappeared after months.

**Conclusion:**

This case demonstrates (a) that congenital lactase deficiency should be considered in cases of severe neonatal diarrhoea, (b) that intestinal biopsies can be avoided in typical cases that are confirmed by genetic testing, and (c) that the associated nephrocalcinosis can be reversed on diet and an appropriate fluid management.

## Background

Congenital lactase deficiency (CLD, OMIM #223000) is a rare but severe gastrointestinal disorder. Characteristic symptoms are osmotic diarrhoea with subsequent dehydration and loss of weight despite adequate caloric intake. Symptoms begin during the first week of life due to the ingestion of lactose-containing breast milk or formula. First descriptions of CLD date back to the late 1950s [1, 2]. CLD is caused by a deficiency of intestinal lactase activity (EC 3.2.1.23) which is the result of mutations of the lactase-phlorizin hydrolase gene (*LCT*) whose 49.3 kb of genomic DNA organized in 17 exons are located on 2q21.3 [3–5]. In Finland, a single *LCT* mutation is highly prevalent (carrier frequency 1/35) with the result that this autosomal-recessive disorder is relatively frequent there. A total of seven different *LCT* mutations have been described so far in Finnish patients [4, 6]. Recently, the first mutations of patients from outside Finland have been found [6, 7]. We present the first genetically confirmed case of congenital lactase deficiency diagnosed in Central Europe based upon detection of a novel mutation within the *LCT* gene.

## Case presentation

**Fig. 1:**
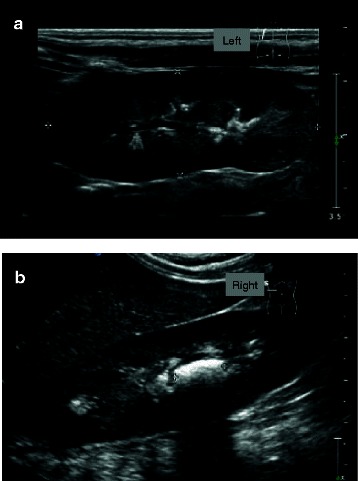
Nephrocalcinosis. Ultrasound of left (**a**) and right (**b**) kidney at time of admission to the hospital. *Note* parenchymal densities partly accompanied by dorsal echo extinction

We present the case of a male newborn of consanguineous parents of Turkish origin living in Germany. Family history was unremarkable, particularly cases of neonatal diarrhoea or unexplained death shortly after birth were not observed. The child was born at term with a weight of 3380 g. He developed 12–15 watery stools per day after breast feeding was started. He was admitted to hospital on postnatal day 17 with a weight loss of 14 % as compared to birth weight and with signs of hypertonic dehydration (Na^+^ 152 mmol/l) and metabolic acidosis (pH 7,29; base excess −11,4 mmol/l, HCO_3_^−^ 13,6 mmol/l, pCO_2_ 29 mmHg). Highly elevated calcium concentrations were measured in blood (4,02 mmol/l) and urine (10,5 mmol/l) and an ultrasound examination of the abdomen showed nephrocalcinosis and, later in the course, nephrolithiasis (Fig. 1).

Intravenous rehydration was started immediately and a glucose-, galactose-, and lactose-free formula was fed. The diarrhoea stopped and the patient’s general condition improved substantially. Therefore, glucose-galactose-malabsorption (GGM) was assumed. However, genetic testing of *SGLT1* (*SLC5A1*) was negative and, as expected, oral ingestion of maltodextrine did not provoke diarrhoea.

**Fig. 2:**
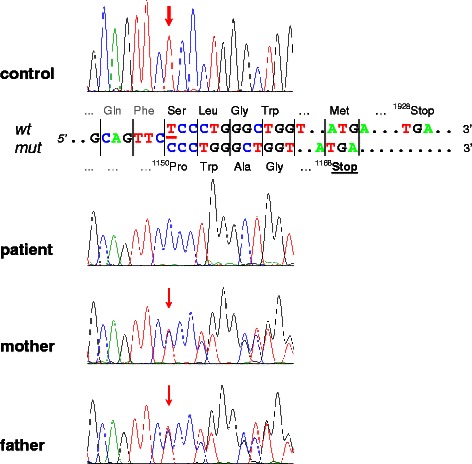
Novel mutation of the *LCT* gene. Results of DNA sequencing of a PCR product containing exon 8 of the *LCT* gene. Presented are chromatographs of the patient, both parents, and a control. Depicted is the region of codon 1150 demonstrating homozygosity for a 1-bp deletion in the patient. Both parents show a heterozygous pattern for this mutation. *wt*, wild-type sequence; *mut*, mutated sequence

Once the patient was fed with lactose, however, he immediately developed watery diarrhoea again, suggesting congenital lactase deficiency. Invasive measures, such as an intestinal biopsy in order to measure lactase activity, were not performed since we saw a dramatic improvement on a lactose-free diet. Instead, genetic testing of the lactase-phlorizin hydrolase gene (*LCT*) was performed revealing homozygosity for a 1-bp deletion in exon 8 (c.3448delT). This mutation predicts a frameshift and a premature termination of translation of the lactase pre-pro-protein (p.S1150Pfs*19) (Fig. 2).

After a lactose-free diet had been introduced and diarrhoea had subsided, the patient gained weight and his psychomotor development was normal. Importantly, hypercalcaemia and hypercalciuria disappeared only within months. Nephrocalcinosis improved slowly, however, several calcium oxalate stones came off with severe pain attacks and, eventually, a last prevesical stone was removed endoscopically at an age of 17 months.

## Discussion

Based on the detection of a novel mutation within the *LCT* gene, we present the first genetically confirmed case of congenital lactase deficiency (CLD) diagnosed in Central Europe. This mutation has not been described in the literature so far but according to its nature, there is no doubt that it is disease-causing. In our patient, we found a small deletion which predicts a frame shift and a truncated protein with loss of the active site, and it is well conceivable that this will alter trafficking to the brush border membrane as already shown for other truncating *LCT* mutations [8]. This is only the third report of *LCT* mutations from outside Finland (Table 1). The first publication on an Italian patient and Turkish siblings [4] was followed by a single compound heterozygous patient from Japan [7, 9]. The fact that this is the very first report on a genetic defect of *LCT* from Central Europe, a region with a traditional interest in this condition [10], confirms that CLD is really rarely encountered here, definitely with a frequency much lower than 1:60,000 as estimated for Finland [3].

**Table 1:** Reported patients with *LCT* mutations

	pt #	ethnic origin		exon	mutation	mutation effect ^c^	
Kuokkanen 2006 [4]	1–27	Finland	homozygous	ex09	c.4170T > A	p.Y1390*	*nonsense (truncating)*
	28–29	Finland	compound heterozygous ^a^	ex14	c.4998_5001delTGAG	p.S1666Kfs*58	*fs (truncating)*
	30	Finland	compound heterozygous ^a^	ex02	c.653_654delCT	p.S218Cfs*6	*fs (truncating)*
	31	Finland	compound heterozygous ^a^	ex03	c.804G > C	p.Q268H	*missense*
	32	Finland	compound heterozygous ^a^	ex09	c.4087G > A	p.G1363S	*missense*
Torniainen 2009 [6]	33	Italian	compound heterozygous	ex07	c.2062T > C	p.S688P	*missense*
			compound heterozygous	ex12	c.4834G > T	p.E1612*	*nonsense (truncating)*
	34	Finland	compound heterozygous ^a^	ex06	c.1692_1696delAGTGG	p.V565Lfs*3	*fs (truncating)*
	35	Finland	compound heterozygous ^a^	ex12	c.4760G > A	p.R1587H	*missense*
	36	Turkish ^b^	homozygous	ex09	c.4087G > A	p.G1363S	*missense*
Uchida 2012 [7]	37	Japanese	compound heterozygous	ex10	c.4419C > G	p.Y1473*	*nonsense (truncating)*
			compound heterozygous	ex16	c.5387delA	p.D1796Afs*18	*fs (truncating)*
**This study 2015**	38	Turkish	homozygous	ex08	c.3448delT	p.S1150Pfs*19	*fs (truncating)*

^a^ compound heterozygosity with c.4170 T > A; ^b^ two siblings; ^c^ fs, frameshift and premature stop (*) of translation

In the past, intestinal biopsies had to be investigated in such cases in order to confirm the suspected diagnosis. Although these biopsies had to be gained via gastroduodenoscopy, an invasive and cost-intensive measure, the gold standard of diagnosis in case of suspected CLD was the Dahlquist method [11] measuring the lactase activity within an intestinal specimen. Our case emphasizes that intestinal biopsies are no longer needed in most patients in whom CLD is suspected. The symptoms of CLD are consistent and can easily be provoked by lactose ingestion; thus, patients with CLD can be reliably selected by a concise clinical investigation. Genetic testing for mutations of the *LCT* gene rather than intestinal enzymatic studies (or even H_2_ malabsorption studies) should be performed whenever CLD is suspected in patients with typical symptoms and a positive resoponse to dietary elimination of lactose. The result of a genetic test describes the basic defect and excludes secondary effects; thus, it is more reliable for genetic counseling and for family planning. This test should be used generously in suspicious cases since Torniainen *et al.* concluded from molecular genetic studies in Finland that the prevalence of CLD might be higher than assumed [6]. As CLD is a severe disease that can easily be treated by dietary means once the correct diagnosis is established, genetic testing for CLD should be considered early by gastroenterologists, neonatologists and paediatricians.

Also we found that CLD is associated with nephrocalcinosis as described previously. Thus, our observation is in line with the report of Saarela *et al.* [12] describing hypercalcaemia already at the time of diagnosis of CLD. Calcium concentrations in blood and excretion with urine normalized only within months after the correction of dehydration and beginning of a lactose-free diet in our case. Our case also confirms that it may take years until problems of nephrocalcinosis and nephrolithiasis fully disappear [12]. Nephrocalcinosis can be due to dehydration and due to chronic metabolic acidosis as it has been described in the context of glucose-galactose-malabsorption [13]. The complete pathogenesis of nephrocalcinosis in CLD has yet remained unclear.

## Conclusions

This case demonstrates (a) that congenital lactase deficiency should be considered in cases of severe neonatal-onset diarrhoea, (b) that in typical cases, responding to lactose elimination from the diet, intestinal biopsies are not the first-line diagnostic steps and should be replaced by genetic testing, and (c) that the associated nephrocalcinosis can be a long-lasting problem despite appropriate rehydration and dietary measures.

## Consent

Written informed consent was obtained from the patient’s parents for publication of this case report and any accompanying images. A copy of the written consent is available for review by the Editor of this journal.

## Abbreviations

CLD: Congenital lactase deficiency
GGM: Glucose-galactose-malabsorption
